# A putative ATP/GTP binding protein affects *Leishmania mexicana* growth in insect vectors and vertebrate hosts

**DOI:** 10.1371/journal.pntd.0005782

**Published:** 2017-07-24

**Authors:** Aygul Ishemgulova, Natalya Kraeva, Jana Hlaváčová, Sara L. Zimmer, Anzhelika Butenko, Lucie Podešvová, Tereza Leštinová, Julius Lukeš, Alexei Kostygov, Jan Votýpka, Petr Volf, Vyacheslav Yurchenko

**Affiliations:** 1 Life Science Research Centre, Faculty of Science, University of Ostrava, Ostrava, Czech Republic; 2 Biology Centre, Institute of Parasitology, Czech Academy of Sciences, České Budejovice (Budweis), Czech Republic; 3 Department of Parasitology, Faculty of Science, Charles University, Prague, Czech Republic; 4 Department of Biomedical Sciences, University of Minnesota Medical School, Duluth, Minnesota, United States of America; 5 University of South Bohemia, Faculty of Sciences, České Budejovice (Budweis), Czech Republic; 6 Integrated Microbial Biodiversity Program, Canadian Institute for Advanced Research, Toronto, Ontario, Canada; 7 Zoological Institute of the Russian Academy of Sciences, St. Petersburg, Russia; 8 Institute of Environmental Technologies, Faculty of Science, University of Ostrava, Ostrava, Czech Republic; 9 Department of Pathology, Albert Einstein College of Medicine, Bronx, New York, United States of America; Universidade Federal de Minas Gerais, BRAZIL

## Abstract

**Background:**

*Leishmania* virulence factors responsible for the complicated epidemiology of the various leishmaniases remain mainly unidentified. This study is a characterization of a gene previously identified as upregulated in two of three overlapping datasets containing putative factors important for *Leishmania*’s ability to establish mammalian intracellular infection and to colonize the gut of an insect vector.

**Methodology/Principal findings:**

The investigated gene encodes **A**TP/GTP binding motif-containing protein related to ***L****eishmania* **d**evelopment **1** (ALD1), a cytosolic protein that contains a cryptic ATP/GTP binding P-loop. We compared differentiation, growth rates, and infective abilities of wild-type and ALD1 null mutant cell lines of *L*. *mexicana*. Loss of ALD1 results in retarded growth kinetics but not defects in differentiation in axenic culture. Similarly, when mice and the sand fly vector were infected with the ALD1 null mutant, the primary difference in infection and colonization phenotype relative to wild type was an inability to achieve maximal host pathogenicity. While ability of the ALD1 null mutant cells to infect macrophages *in vitro* was not affected, replication within macrophages was clearly curtailed.

**Conclusions/Significance:**

*L*. *mexicana* ALD1, encoding a protein with no assigned functional domains or motifs, was identified utilizing multiple comparative analyses with the related and often experimentally overlooked monoxenous flagellates. We found that it plays a role in *Leishmania* infection and colonization *in vitro* and *in vivo*. Results suggest that ALD1 functions in *L*. *mexicana*’s general metabolic network, rather than function in specific aspect of virulence as anticipated from the compared datasets. This result validates our comparative genomics approach for finding relevant factors, yet highlights the importance of quality laboratory-based analysis of genes tagged by these methods.

## Author summary

Leishmaniases are human parasitic diseases caused by several species of insect-transmitted *Leishmania*. The pathogenic genus *Leishmania* has close relatives that also colonize the guts of insects but are not able to infect mammalian cells. When the genes and gene expression profiles of pathogenic *Leishmania* are compared to those of their nonpathogenic relatives, some genes are either only present or expressed at higher levels in the pathogenic parasites. Protein products of these genes are likely to be involved in the parasites‘ ability to colonize two hosts. Identification of their function will lead to a better understanding of leishmaniases and may reveal potential new drug targets to combat disease. Experiments here are aimed at characterizing a gene identified in this manner. The sequence of the gene’s protein product was examined to identify motifs that gave clues to its function, and the protein itself was determined to be located in the cytosol of *Leishmania mexicana* cells. When the gene was deleted from *L*. *mexicana*, it compromised *L*. *mexicana*’s ability to grow quickly in culture, to achieve maximal levels of parasitemia in mice, and to achieve maximal parasite loads in insects.

## Introduction

The genus *Leishmania* unites parasitic protozoa of the family Trypanosomatidae causing leishmaniases, diseases affecting human and animal populations mainly in tropical and subtropical regions. Clinical manifestations vary from spontaneously healing skin lesions to potentially fatal visceral organ failures. Leishmaniases are a global human health problem with over 350 million people at risk [1,2]. Approximately two dozen species of *Leishmania* pathogenic to humans have been described [3]. These are mainly transmitted by the bite of female phlebotomine sand flies [4,5]. Knowledge of factors affecting *Leishmania* growth and development in both sand fly and vertebrate hosts is fundamental to understanding the complicated epidemiology of leishmaniases and the development of new treatments and preventatives.

The life cycle of *Leishmania* consists of two replicative developmental stages—extracellular promastigotes, which multiply and develop within the sand fly's alimentary tract, and intracellular amastigotes, multiplying within the phagolysosomal vacuoles of their vertebrate host's phagocytic cells [6,7]. All differentiation events are characterized by dramatic changes in parasite morphology, composition of cell surface glycolipids, gene expression, and other cellular features [8–10]. Gene products involved in either differentiation or infection maintenance in both hosts are potential virulence factors, i.e. entities determining pathogenicity.

Comparative genomics and transcriptomics provide promising new tools to identify crucial factors affecting parasite development in its hosts. For example, analysis of ploidy among *Leishmania* spp. revealed that the tetrasomic chromosome 30 in *L*. *mexicana* is highly enriched for amastigote-specific genes, thus linking chromosome number and adaptation to the vertebrate host in *Leishmania* [11]. Several orthologs of the genes located on the chromosome 30 in *L*. *mexicana*, e.g. amino acid transporters (*LmxM*.*30*.*0330*, *LmxM*.*30*.*0571*, *LmxM*.*30*.*0870*, *LmxM*.*30*.*1820*), tryparedoxin (*LmxM*.*30*.*1960*), aquaglyceroporin 1 (*LmxM*.*30*.*0020*), and one member of the ABC transporter superfamily (*LmxM*.*30*.*1290*) were already implicated in virulence [12–14].

Another example stemmed from the generally accepted view that dixenous (shuttling between insect vector and vertebrate host) *Leishmania* parasites emerged within the clade of monoxenous (one host) insect trypanosomatids of the subfamily Leishmaniinae [15–17]. Our previous OrthoMCL analysis on a dataset of 27 annotated trypanosomatid genomes delineated 99 orthologous groups (OGs) gained at the basal node of *Leishmania* [18]. The timing of their acquisition suggests that they may be critical for dixeny, and indeed, this set includes several known virulence factors. As is typical for the Trypanosomatidae, 87 of 99 OGs correspond to proteins of unknown function, highlighting the need for future gene-focused functional studies.

In a search for new candidates for *Leishmania* virulence factors we examined gene expression in three different datasets [18]: i) genes up-regulated at elevated temperatures in *Leptomonas seymouri* as a pre-adaptation to dixeny; ii) genes up-regulated in a virulent isolate of *Leishmania major* LV561 compared to an attenuated isolate; and iii) genes overexpressed in the virulent metacyclic promastigotes and amastigotes of *L*. *mexicana* M379. Twenty OGs were found to be shared by at least two of these datasets, eleven of which had known virulence factor annotations, while the remaining nine represented proteins of unknown function that are potentially involved in *Leishmania* virulence. Of these nine, we selected the only gene located on the tetrasomic amastigote-gene enriched chromosome 30 of *L*. *mexicana* (*LmxM*.*30*.*2090*). Its orthologs were up-regulated in *Leptomonas seymouri* at 35°C and in *L*. *mexicana* amastigotes [18]. Here we investigate the putative function(s) of this protein using *in silico* and *in vitro* approaches and analyzed its role in *Leishmania* development in insect vectors and mouse hosts.

## Materials and methods

### *In silico* analyses

Protein sequences encoded by *LmxM*.*30*.*2090* and its orthologs were extracted from the TriTrypDB v.9.0 [19] and aligned using Muscle v.3.8.31 with default parameters [20]. Alignment was visualized using Jalview v.2.9 [21].

### *Leishmania* axenic cultivation, growth, and differentiation

*Leishmania mexicana* (isolate MNYC/BZ/62/M379) culture was maintained in M199 medium (Sigma-Aldrich, St. Louis, USA) supplemented with 2 μg/ml Biopterin (Sigma-Aldrich), 2 μg/ml Hemin (Jena Bioscience GmbH, Jena, Germany), 25mM HEPES, 50 units/ml of penicillin, 50 μg/ml of streptomycin and 10% Fetal Bovine Serum, FBS (all from Life Technologies, Carlsbad, USA) at 23°C. Both ALD1 knock-out (KO) and WT *L*. *mexicana* were passaged through insects and mice prior to *in vitro* analyses. Growth kinetics comparison was performed for 6 days from the starting density of 5 x 10^5^ parasites per ml. Cell numbers were counted using a hemocytometer every 48 hours as described previously [22] in four biological replicates.

*In vitro* differentiation was performed as described elsewhere by varying pH and temperature [23] with modifications. In particular, procyclic and metacyclic promastigotes were collected 8 hours apart for WT and KO lines on days 3 and 11, respectively. WT amastigotes were collected on day 18, while KO amastigotes were collected on day 21 of the experiment based on the assessment of cell morphology in culture. For normalization, expression values of *LmxM*.*07*.*0510* (gene encoding a 60S ribosomal protein L7a) and *LmxM*.*36*.*1140* (gene encoding a short chain 3-hydroxyacyl-CoA dehydrogenase) were used [24]. Quantitative PCR analysis (RT-qPCR) was performed as described previously [25]. Primer sequences for RT-qPCR are listed in the S1 Table.

### Genetic manipulations in *Leishmania mexicana*

To ablate *LmxM*.*30*.*2090* in *L*. *mexicana*, both alleles were sequentially replaced with selectable markers for Nourseothricin (Sat) and Hygromycin (Hyg). Targeting constructs were generated by fusion PCR [26]. In the first round of PCR, 5' and 3' arms of homology were amplified from the *L*. *mexicana* genomic DNA using primers A/B (or C) and D (or E)/F, respectively (S1 Table). The ORFs of the Sat and Hyg-resistance genes were amplified from the plasmids pF4T7polNLS1.4sat and pF4TR1.4hyg [27] using primers SAT_5'f/ SAT_3'r and Hyg_5'f/ Hyg_3'r (S1 Table). In the fusion PCR reaction, 5' and 3' arms of homology were combined with either Sat or Hyg-resistance gene and amplified with nested primers G and H (S1 Table). *L*. *mexicana* promastigotes were transfected with 5 μg of the targeting constructs as described previously using BTX ECM 630 electroporator (Harvard Apparatus, Inc, Holliston, USA) [28]. The first allele knockout cell line (*LmxM*.*30*.*2090*^+/-^) was isolated in complete M199 medium containing 100 μg/ml of Sat (Jena Bioscience GmbH). The *L*. *mexicana LmxM*.*30*.*2090*^-/-^ (knock-out, KO) clones were selected on solid M199 medium supplemented as above with additional 100 μg/ml of Hyg. Correct integration was confirmed by PCR on genomic DNA with specific primers and by Southern blot [29]. In brief, total genomic DNA was isolated using DNeasy Blood & Tissue Kit (Qiagen, Hilden, Germany), digested with *Nco* I overnight, separated on 0.75% agarose gel, and transferred to a Zeta-Probe blotting membrane (Bio-Rad, Hercules, USA). Blots were blocked and hybridized with ^32^P-labeled PCR probes for Sat, Hyg, 5' UTR-, 3' UTR-, and ORF of *LmxM*.*30*.*2090* gene. The following primers (S1 Table) were used to amplify probes: SBp_SAT_f and SBp_SAT_r (for Sat), SBp_Hyg_f and SBp_Hyg_r (for Hyg), SBp_*LmxM*.*30*.*2090*_5'f and SBp_*LmxM*.*30*.*2090*_5'r (for 5' UTR), SBp_*LmxM*.*30*.*2090*_3'f and SBp_*LmxM*.*30*.*2090*_3'r (for 3' UTR), SBp_*LmxM*.*30*.*2090*_f and SBp_*LmxM*.*30*.*2090*_r (for *LmxM*.*30*.*2090* ORF). Probes were labeled with radioactive ^32^P using the DecaLabel DNA Labeling kit (ThermoFisher Scientific, Waltham, USA). PCR confirmation of the correct integration was performed using primers pairs SBp_Hyg_f—SBp_Hyg_r (expected size KO 0.3 kb), Hyg190_f—Hyg_3’r (expected size KO 0.8 kb), A—SBp_Hyg_r (expected size KO 1.7 kb), SBp_SAT_f—SBp_SAT_r (expected size KO 0.3 kb). The complete ablation of the *LmxM*.*30*.*2090* gene was confirmed with primers pairs A—SBp_*LmxM*.*30*.*2090*_r (expected size wild type, WT 2.0 kb) and *LmxM*.*30*.*2090*_f–*LmxM*.*30*.*2090*_r (expected size WT 0.25 kb). See S1 Table for primer sequences.

For localization studies, the *LmxM*.*30*.*2090* gene was tagged with HA_3_ and expressed from the 18S rRNA locus of *L*. *mexicana*. The open reading frame was amplified from the genomic DNA using primers 30.2090_*Nco*I_f—30.2090_3xHA_*Not*I_r (S1 Table). The HA_3_ tag was included in the reverse primer. The amplified fragment was cloned into pLEXSY-sat2 (Jena Bioscience GmbH). Five μg of the resulting plasmid were linearized with *Swa*I and transfected as above. *LmxM*.*30*.*2090*-HA_3_ cell line was isolated in complete M199 medium containing 100 μg/ml of Sat. PCR confirmation of the correct integration was performed using primers SSU_dir and SBp_*LmxM*.*30*.*2090*_r (expected size 1.8 kb).

### Confocal immunofluorescence microscopy

*Leishmania mexicana* expressing HA-tagged *LmxM*.*30*.*2090* promastigotes were incubated with 100 nM MitoTracker Red CMXRos (ThermoFisher Scientific) in M199 medium according to the manufacturer's instructions. Promastigotes were fixed in 1% paraformaldehyde, immobilized on the polylysine-coated coverslips, permeabilized with 0.1% Triton X100 in PEM buffer (100 mM PIPES pH 6.9; 1 mM EGTA; 100 μM MgSO_4_) and blocked in PEMBALG buffer (PEM buffer; 1% BSA; 0.5% cold water fish skin gelatin; 100 mM lysine). To detected HA-tagged ALD1, primary rat anti-HA antibodies (Roche Diagnostics GmbH, Mannheim, Germany) and Alexa Fluor 488—conjugated secondary anti-rat antibodies (Life Technologies) were used. The coverslips were mounted on slides using VECTASHIELD anti-fade mounting medium with DAPI (Vector Laboratories, Burlingame, USA) and observed with a Leica TCS SP8 WLL SMD-FLIM inverted confocal microscope (Leica Microsystems, Wetzlar, Germany). Images were processed in Fiji Image J v. 2.0.0 [30].

### Bone marrow-derived macrophages culture and infection

Bone marrow-derived macrophages were differentiated for 7–9 days from the precursor cells of BALB/c mice in the presence of 20% L929 fibroblast cell culture supernatant as a source of macrophage-colony stimulating factor. Differentiated macrophages were cultivated in complete RPMI-1640 medium containing 10% FBS, 1 x PenStrep solution, 2 mM of L-glutamine (all from Sigma-Aldrich), and 50 μM of β-mercaptoethanol at 37°C with 5% CO_2_. To assess infection in macrophages, cells (5 x 10^4^ per well) were plated on Lab-Tek chamber slides (ThermoFisher Scientific) and infected with WT or KO *L*. *mexicana* promastigotes freshly passaged through the mice, at a parasite to macrophage ratio of 5:1. Cells remained either unstimulated in complete RPMI 1640 medium or were stimulated 2 hours post infection (p.i.) with 50 U/ml of IFN-γ (Bio-Rad) and 500 ng/ml of LPS (Sigma-Aldrich). Slides were stained with Giemsa at 4 hours, 72 hours, and 6 days p.i. and the percent of infected macrophages and parasite load were counted from four biological replicates with two technical replicates each (400 cells per condition).

### Nitrite and arginase activity analyses

To measure NO production and arginase activity, 5 x 10^4^ macrophages per well were plated in the flat bottom Costar 96-well plates (Sigma-Aldrich) and infected as above. After 72 h incubation, the supernatant and cell lysate (in quadruplicates) were used for nitrite and urea analysis, respectively. The accumulation of NO_2_^-^ was determined by Griess reagents, the arginase activity was analyzed by measuring the conversion of L-arginine to urea, as previously described [31].

### Infection of insects

To compare the development of *LmxM*.*30*.*2090* null mutant (KO) and WT of *L*. *mexicana* in sand flies, three independent experiments were performed. Laboratory colony of *Lutzomyia longipalpis* (Jacobina, Brazil) was maintained at 26°C under standard conditions as described previously [32]. Sand fly females were fed through a chick skin membrane on a suspension of heat-inactivated rabbit blood containing 10^6^ promastigotes per ml. Blood-fed females were separated and maintained at 26°C. On days 1–2 and 7 post infection (d.p.i) females were checked for localization and intensity of infection with light microscope. Infection was graded as light (< 100 parasites/gut), medium (100–1,000 parasites/gut), and heavy (> 1,000 parasites/gut) as previously described [33].

Smears of dissected and examined guts 7 d.p.i. were air dried, fixed by methanol, stained by Giemsa (Sigma-Aldrich), examined under the Olympus BX51 light microscope equipped with a DP72 CCD camera (Olympus, Tokyo, Japan). Morphometric parameters (length and width of the cell body, as well as length of the flagella) of 540 randomly selected promastigotes from 9 females/smears per each group were measured using QuickPHOTO micro v. 3.0 (Promicra, Prague, Czech Republic). Three main morphotypes were distinguished and categorized as described previously [34] with the slight modifications: i) long nectomonads: body length ≥ 12 μm; ii) short nectomonads (= leptomonads): body length <12 μm; and iii) metacyclic promastigotes: body length ≤ 8 μm and, simultaneously, flagella/body length ratio > 1.5. Results were evaluated using Statistica v. 6.1 (Quest, Aliso Viejo, USA).

### Quantitative PCR analysis of parasites from insects

To quantify the numbers of *Leishmania* parasites in the guts of female sand flies on days 1 and 7 p.i., RT-qPCR with *Leishmania* kinetoplast DNA-specific primers was performed as described before [33]. Total DNA from infected females was extracted using a High Pure PCR Template Preparation Kit (Roche Diagnostics) according to the manufacturer's protocol. Log-transformed data were evaluated using Statistica v. 6.1.

### Mice infection

For mice infections, sand fly females were infected with *L*. *mexicana* WT or KO strains as described above and checked for the presence and localization of parasites 8–9 d.p.i. Pools of freshly dissected thoracic midguts (TM), with colonized stomodeal valve and high parasite density, were homogenized in sterile saline solution. Immediately, 5 μl of the suspension (from ~ 10 sand fly TMs) was intra-dermally injected into the ear pinnae of a ketamin/xylazin anesthetized BALB/c mouse. Groups of four BALB/c mice of each studied group (WT vs. KO) were used in two independent experiments. Disease development was monitored weekly. Mice were sacrificed at the 15^th^ week (experiment 1–4 mice per each group) or 13^th^ week (experiment 2–4 mice per each group) p.i. and infected ears were used for both *Leishmania* re-isolation by cultivation and quantification of parasite infestation by RT-qPCR as described above.

#### Ethics statement

Animals were maintained and handled in the animal facility of Charles University in Prague in accordance with institutional guidelines and Czech legislation (Act No. 246/1992 and 359/2012 coll. on protection of animals against cruelty in present statutes at large), which complies with all relevant European Union and international guidelines for experimental animals. All the experiments were approved by the Committee on the Ethics of Laboratory Experiments of the Charles University in Prague and were performed under permission No. MSMT-31114/2015–13 of the Ministry of the Environment of the Czech Republic. Investigators are certificated for experimentation with animals by the Ministry of Agriculture of the Czech Republic. All efforts were made to minimize the number and the suffering of experimental animals during the study.

## Results

### *LmxM*.*30*.*2090* encodes a cytosolic putative ATP/GTP-binding protein

TriTryp database annotation of the *LmxM*.*30*.*2090* [19] revealed no conserved motifs or domains for the predicted 382 amino acid (aa) long protein. However, we have previously shown that affiliation to protein families can be identified by manual visual analysis [35], so we inspected the sequence further. DELTA-BLAST search with default parameters [36] produced no known protein hits, but gave high E-value (range from 10^−7^ to 10^−32^) hits to smaller regions of annotated proteins with 23–32% identity. Many of these contained ATP-binding folds such as bacterial UvrB helicase [37] with its classical P-loop [38]. While the putative protein encoded by *LmxM*.*30*.*2090* does not resemble a helicase, visual inspection revealed sequences resembling the Walker A and B motifs of ATP/GTP binding P-loops [39,40]. We named this protein ALD1 (**A**TP/GTP binding motif-containing protein related to ***L****eishmania* **d**evelopment **1**) (S1 Fig).

**Fig 1:**
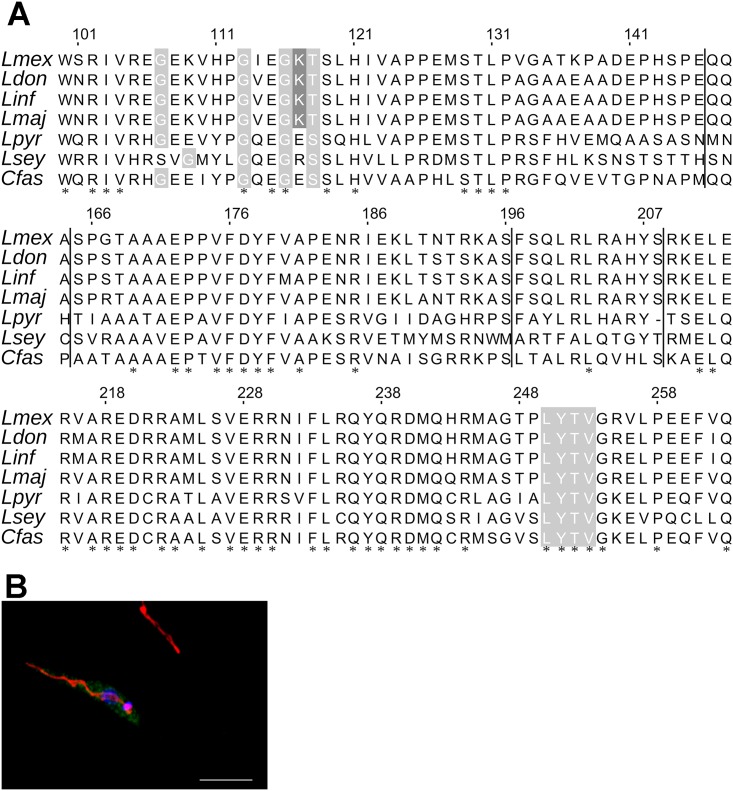
ALD1 primary structure and localization. A, Sequence alignment of the *L*. *mexicana* ALD1 protein and its orthologs found in the subfamily Leishmaniinae. All the columns containing insertions relative to the *L*. *mexicana* protein sequence are hidden and the sites of such insertions are shown with black vertical lines. The Walker motifs A (aa 113–118) and B (aa 250–260) are highlighted in grey; crucial residue in the P-loop represented by K117 of the motif A is shown in dark grey. The numbers on top correspond to the *L*. *mexicana* protein. *Lmex* = *Leishmania mexicana*; *Ldon* = *Leishmania donovani*; *Linf* = *Leishmania infantum*; *Lmaj* = *Leishmania major*; *Lpyr* = *Leptomonas pyrrhocoris*; *Lsey* = *Leptomonas seymouri*; *Cfas* = *Crithidia fasciculata*. B, Cytoplasmic localization of HA-tagged ALD1 analyzed by confocal immunofluorescence microscopy with anti-HA antibodies and Alexa Fluor 488—conjugated secondary antibodies (green). The nuclear and kinetoplast DNAs were stained with DAPI (blue), and mitochondria were stained with MitoTracker Red CMXRos (red). Scale bar is 5 μm.

Using the current canonical definitions of GxxxxGKT/S for Walker A and ΦΦΦΦD for Walker B (Φ denotes hydrophobic aa), ALD1 contains no P-loop. Only by comparison of ALD1 with the original alignment, which included additional conserved sites and alternative site spacing [39] did we observe a P-loop in ALD1 (Fig 1A). ALD1 still lacks an important Mg^2+^-binding D residue in the Walker B domain (substituted by G), but this domain is less conserved and some P-loops lack it entirely [40]. The putative Walker B domain we identified in ALD1 may be vestigial and another D (or E) residue from the sequence may fulfill this role; there are 4 of them in the 20 aa following the Walker B hydrophobic patch in Fig 1. A *Plasmodium falciparum* study demonstrates that significant sequence and spacing deviations are to be expected in the P-loop-containing proteins in parasitic protists [41].

*In silico* secondary structure inference of ALD1 by PredictProtein [42] revealed a region from aa 193 to 220 strongly predicted as alpha helical (labeled H(p) in S1 Fig). A larger region (dotted area in S1 Fig) modeled to alpha-helical domains of a range of proteins in Swiss-Model [43] and was also predicted as alpha-helical by PredictProtein with less confidence. Protein families with alpha-helical domains preceding the Walker B domain include the ABC transporter/SMC family, AAA+ superfamily, and MCM proteins [44]. The transmembrane domain prediction programs indicated that ALD1 is unlikely to be a membrane protein. In agreement with this, direct analysis of the tagged protein localization by confocal microscopy revealed it to be cytoplasmic (Fig 1B).

If the cryptic ALD1 P-loop is important for *Leishmania’s* ability to infect multiple hosts, there may be differences in this domain between dixenous *Leishmania* and monoxenous Leishmaniinae. Indeed, this is the case (Fig 1A). The intact Walker A motif (GxxxxxGxxGKT/S) was documented only in *Leishmania* spp. Similar sequence motifs were present in monoxenous *Leptomonas* and *Crithidia* spp., but the most vital residue in the P-loop (K117 of the motif A of *L*. *mexicana* ALD1 [40]) was substituted by either E or R residues in these orthologs (Fig 1A). In summary, ALD1 is a cytosolic protein that most likely requires nucleotide binding for a function in *Leishmania* not shared with its orthologs in related monoxenous flagellates.

### Genetic ablation of *LmxM*.*30*.*2090* encoding ALD1

**Fig 2:**
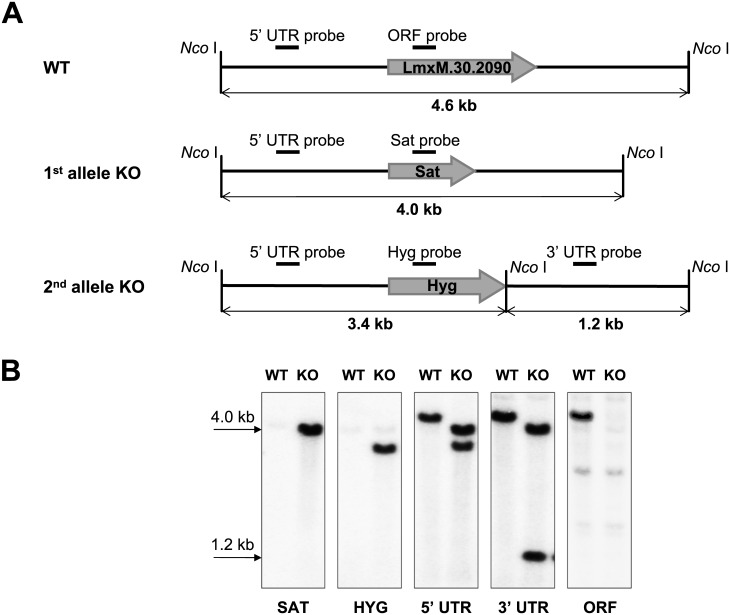
Ablation of the *LmxM*.*30*.*2090* in *L*. *mexicana*. A, Schematic representation of the WT and recombined alleles. Annealing positions of the probes and expected fragment sizes are shown. B, Southern blot analysis of the *Nco* I digested *L*. *mexicana* genomic DNA of the WT and ALD1 KO strains with Sat, Hyg, 5' UTR, 3' UTR, and *LmxM*.*30*.*2090* ORF probes.

ALD1 was initially identified as potentially related to adaptation to a dixenous lifestyle [18]. We therefore hypothesized that ALD1 may be involved in parasites’ ability to infect insect or vertebrate hosts. To examine the role of ALD1 in *Leishmania* virulence, we established *LmxM*.*30*.*2090*^-/-^ *L*. *mexicana* strains by homologues recombination. Genes for Sat or Hyg resistance were sequentially transfected into promastigotes replacing all wild type alleles. Southern blot analysis (Fig 2) confirmed replacement of the *LmxM*.*30*.*2090* alleles with resistance markers and generation of the complete null-mutant. Additional confirmations by PCR, qPCR and RT-qPCR are presented in S2 Fig. Of note, chromosome 30 of *L*. *mexicana* is tetraploid. Although only two selectable markers were used, all four alleles were successfully ablated. Molecular mechanisms behind this phenomenon need to be investigated further. The strain, lacking all copies of ALD1, was designated “ALD1 KO”, and the parental cell line “WT”.

### Growth kinetics and differentiation of ALD1 KO and WT *Leishmania* strains *in vitro*

**Fig 3:**
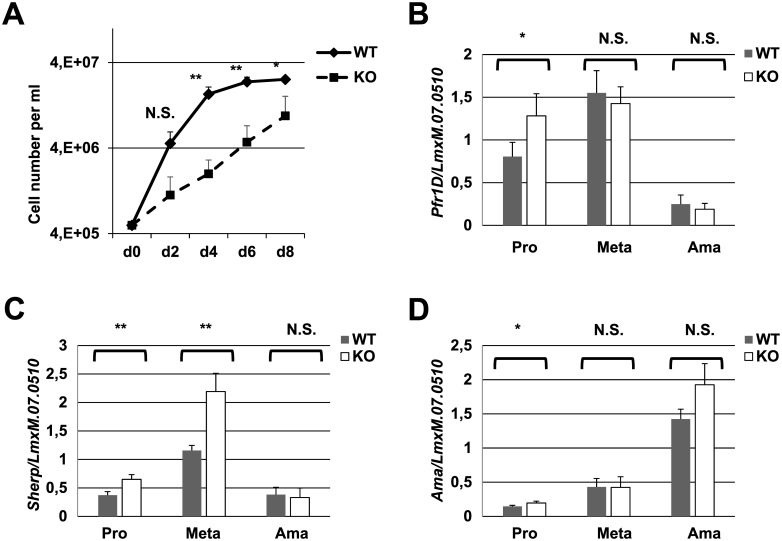
Growth kinetics and differentiation of *Leishmania* strains *in vitro*. A, Growth curves of WT and ALD1 KO *L*. *mexicana*. B, C, D, quantification by RT-qPCR of *Pfr1D*, *Sherp*, and *Amastin* gene expression used as markers for promastigotes (both pro- and metacyclics), metacyclics, and amastigotes, respectively. Data are from four independent biological replicates. The error bars indicate standard deviations. *LmxM*.*07*.*0510* was used for normalization. T-test statistical values are symbolized by asterisks: * (≤ 0.05), ** (≤ 0.01), or N.S. (not statistically significant).

We first investigated the effect of *LmxM*.*30*.*2090* ablation on *L*. *mexicana* growth independent of a host infection by comparing cell division kinetics of WT and ALD1 KO strains *in vitro*. To exclude the negative effect of continuous cultivation [45], procyclic promastigote cultures were started from *Leishmania* cells that had previously been passaged first through insects and then mice. Cell growth monitored every 48 hours in a continuously-growing culture revealed that *Leishmania* lacking ALD1 grew significantly slower than their WT counterparts *in vitro* (Fig 3A). While WT cells exhibited a typical culture growth profile where initial rapid growth slowed by day 4 and reached a plateau at approximately days 6–8, ALD1 KO cells grew slowly and remained in the linear growth phase for the duration of the experiment. This result indicates that the role of ALD1 may not be confined to the ability to establish an infection in either an insect or mammalian host.

*Leishmania’s* ability to complete the life cycle is key for survival and infectivity. Facilitating the study of *Leishmania* cell cycle progression, the transition from procyclic- through metacyclic promastigote stage to the amastigote stage can be reproduced in axenic culture [23]. To discover whether loss of ALD1 resulted in any gross defects in these transitions, both WT and ALD1 KO of *L*. *mexicana* were differentiated *in vitro* and compared for indications of incomplete or altered transitions between life stages. Morphological examination of *Leishmania* cells *in vitro* demonstrated normal development of both strains, albeit with differences in timing (see Materials and methods for details) that are likely due to the differences in relative replication rates as presented in Fig 3A. Also indicating normal ALD1 KO development *in vitro* is qRT-PCR expression analysis of previously identified stage-specific markers (*Pfr1d* expressed in procyclic and metacyclic promastigotes, *Sherp* expressed in metacyclic promastigotes, and *Amastin* expressed in amastigotes). Expression of these genes was analyzed for each strain upon the culture reaching the indicated life stage (Fig 3B, 3C and 3D) and showed no major defect in ALD1 KO. We documented slight, yet statistically significant, increase in levels of *Pfr1d* and *Sherp* in ALD1 KO procyclic and metacyclic promastigotes, respectively, compared to the wild type *L*. *mexicana*. A possible explanation for this is compensatory: the slower dividing mutant cells must possess higher levels of these gene products to achieve the same stage of differentiation as their wild type counterparts. In conclusion, our axenic culture studies indicate that while ALD1 is essential for optimal growth of *L*. *mexicana* promastigotes, its absence does not present obvious barriers to the life stage transitions we can approximate in culture.

### Infection of sand flies with ALD1 KO cells

**Fig 4:**
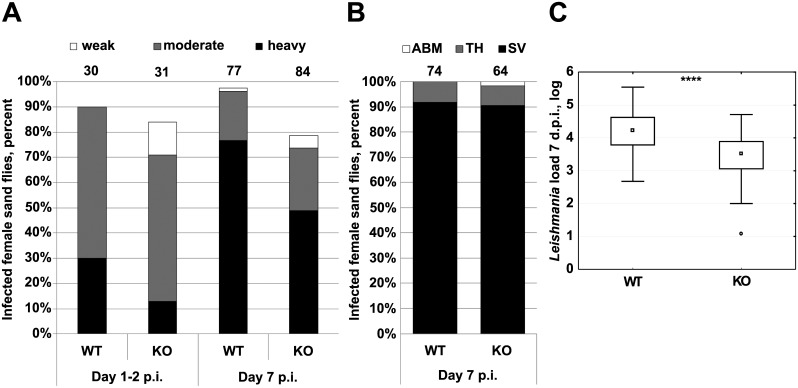
Experimental infection of *Lutzomyia longipalpis* with WT and ALD1 KO *Leishmania mexicana*. A, Intensity of infection was assayed on days 1–2 and 7 p.i. and defined as light (less than 100 promastigotes, white bar), moderate (100–1,000 promastigotes, grey bar), or heavy (over 1,000 promastigotes, black bar) depending on the number of parasites per gut. Numbers above each bar indicate the number of dissected females. B, Localization of *L*. *mexicana* in the insect gut 7 d.p.i. ABM, abdominal midgut; TH, thoracic midgut, SV, stomodeal valve. Numbers above each bar indicate the number of dissected females. C, Quantitative PCR analysis of the *L*. *mexicana* load in the insect gut 7 d.p.i. Boxplots in C are from three independent biological replicates and show 1st quartile, median, 3rd quartile, and 1.5 x interquartile range values. T-test statistical value is symbolized by asterisks: **** (≤ 0.0001).

Since axenic ALD1 KO promastigotes replicate more slowly than WT cells, it is important to determine whether the loss of ALD1 impacts *L*. *mexicana*’s ability to infect its insect host where they also replicate extracellularly. In sand flies, the development of *L*. *mexicana* WT and ALD1 KO was studied on days 1–2 and 7 post infections in three independent biological replicates. Both strains established infection well in *Lu*. *longipalpis*. Percentages of infected females were over 95% for the WT and about 80% for the ALD1 KO strain on day 7 p.i. (Fig 4A). This difference was statistically significant (p = 0.003), the percentage of infected females remained high in both groups tested. As shown in Fig 4B, no differences were observed in the localization of parasites at late-stage infections (7 d.p.i.) and stomodeal valve colonization was observed in the majority (> 90%) of these infected females in both tested groups. On the other hand, the quantitative PCR analysis revealed significantly lower numbers of parasites in ALD1 KO-infected sand flies compared to the WT infected group in both, 1 d.p.i. (F_(1; 38)_ = 16.56; p < 0.001) as well as 7 d.p.i. (F_(1; 134)_ = 56.11; p < 0.0001) (Fig 4C). This trend can also be observed in Fig 4A, where the parasite burden was estimated directly by microscopic observation rather than indirectly by PCR. The slower growth of ALD1 KO strain *in vitro* (Fig 3A), and lack of any differences in colonization suggest that the differences in parasite burden are most likely due to slower growth of ALD1 KO in the insect once the infection is started.

Finally, morphological analysis of parasite cells obtained from infected female flies 7 d.p.i. (540 cells per strain) revealed that metacyclic promastigotes, the sand fly life stage capable of infecting macrophages, represented ~19% of all forms in both ALD1 KO and WT infections. Remaining two forms, long nectomonads and the developmental precursor of metacyclic promastigotes, short nectomonads (leptomonads), constituted 35% (WT) / 17% (ALD1 KO) and 46% (WT) / 64% (ALD1 KO) of flagellates, respectively. As short nectomonads develop from the long form, the higher proportion of short nectomonads in ALD1 KO may be due to enhanced transformation of long to short nectomonads. Interestingly, the stable percentage of metacyclic promastigotes in WT and KO (despite the differing precursor form ratios) implies the existence of a yet unknown regulatory mechanism for maintaining metacyclic promastigote abundance in sand flies. This morphological analysis represents the single clear relationship we found between ALD1 and *Leishmania* developmental transitions.

### Infection of mice with ALD1 KO cells

To determine whether the ALD1 KO *in vitro* parasites' growth phenotypes would result in differences in parasites' infectivity in a mammalian host, BALB/c murine infections with ALD1 KO or WT strains were compared. Infection with both strains resulted in clinical symptoms in all inoculated mice. The mean size of nodular lesions at the end of the experiment in WT and ALD1 KO-infected mice was also similar: 8.2 (± 2.5) and 6.9 (± 1.1) mm, respectively. Quantitative PCR analysis on infected ears from mice sacrificed 13 and 15 weeks p.i. showed high numbers of *Leishmania* parasites in both groups (S3 Fig). However, the parasite load was slightly but significantly (F_(1; 14)_ = 5.28; p = 0.037) higher in the WT control- compared to the ALD1 KO-infected mice. From this analysis in BALB/c mice, we conclude that *L*. *mexicana* lacking ALD1 is able to generate a symptomatic mammalian infection. Importantly, however, ALD1 is necessary for achieving maximal *L*. *mexicana* parasite load, a finding that may be related to its reduced replication rate in an extracellular environment.

### Macrophage infection *in vitro*

**Fig 5:**
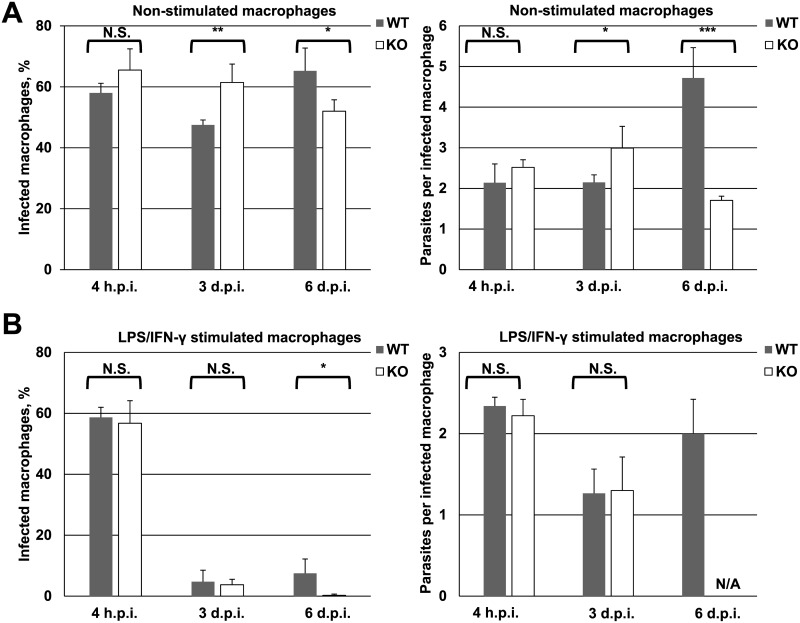
Macrophage infection *in vitro*. The percentage of infected macrophages and the average number of parasite per infected cell were calculated for WT and KO *L*. *mexicana* infecting non-stimulated (A) or LPS/IFN-γ stimulated (B) primary murine macrophages. Data are summarized from four independent biological replicates (400 cells per condition). The error bars indicate standard deviations. T-test statistical values are symbolized by asterisks: * (≤ 0.05), ** (≤ 0.01), *** (≤ 0.001), or N.S. (not statistically significant). N/A, not available.

To parse the source of the compromised mammalian parasite loads in ALD1 KO infections, we asked whether defects in ALD1 KO virulence existed in infections of cultured primary murine macrophages. We first tested the ALD1 KO strain’s ability to infect unstimulated macrophages at three time points, early infections (4 h.p.i.), established infections (3 d.p.i.), and later in infection (6 d.p.i.). While we observed some differences in percentages of macrophages infected between ALD1 KO and WT, the magnitude of the differences were quite small (~10–15%) and trended both up and down, suggesting that ALD1 KO did not possess a gross defect in the ability to infect macrophages or differentiate to the amastigote stage in the context of a cellular infection (Fig 5A, left panel). Similarly, at the first two time points very little difference in number of amastigotes per cell was observed. However, at the 6 d.p.i. a robust increase in number of amastigotes per cell was observed in the WT, whereas numbers failed to go up in the ALD1 KO, pointing to a defect in replication once intracellular infection was established (Fig 5A, right panel).

We then examined whether results of unstimulated macrophage infections would be reflected in classically activated macrophages stimulated with LPS/IFN-γ. In this case, at later time points there are far fewer infected macrophages in both WT and ALD1 KO (Fig 5B, left panel). As macrophages stimulated in this manner typically produce nitric oxide (NO) which is toxic for parasites, such a result is not unexpected. It did however prompt us to ask whether macrophage production of NO (a marker of classically activated macrophages), or urea (a marker of alternatively activated macrophages) [46] differed in cells infected with ALD1 KO and WT *L*. *mexicana* strains. We found no significant differences between the ALD1 KO and WT infected non-stimulated or IFN-γ+LPS stimulated macrophages regarding the production of NO (F_(1, 61)_ = 1.74, p = 0.19^non-stimulated^; F_(1, 61)_ = 0.68, p = 0.41^IFN-γ+LPS^) or urea (F_(1, 61)_ = 0.27, p = 0.61^non-stimulated^; F_(1, 61)_ = 3.76, p = 0.07^IFN-γ+LPS^).

Because numbers of infected cells were so low, it was more difficult to arrive at firm conclusions regarding the effect of ALD1 ablation on stimulated macrophage infection. However, we noticed that at 4 h.p.i. similar numbers of macrophages were infected, suggesting that ALD1 KO harbored no defects in initial infection and transition to the amastigote stage. The only differences between WT and ALD1 KO infections are again observed 6 d.p.i. While the percentage of infected cells in WT at 6 d.p.i. was quite low (5%), there was almost a complete absence of infected cells in ALD1 KO, with only one cell containing a single amastigote observed (Fig 5B, right panel).

We interpret this result in the light of activated macrophages’ enhanced ability to kill *Leishmania*. In WT *L*. *mexicana* infections, at 6 d.p.i. percentages of infected cells are barely being maintained despite the amastigotes’ normal replication rate. It would then follow that in the ALD1 KO, a failure to replicate intracellularly would result in a loss of infection overall at this time point. In summary, we concluded that WT and ALD1 KO *L*. *mexicana* differ in their ability to survive in primary murine macrophages *in vitro*, and that this difference lies in amastigotes‘ ability to replicate intracellularly.

## Discussion

We have identified ALD1 as a novel factor affecting the fitness of *L*. *mexicana*. Our investigation utilized an ALD1 null mutant for *in vitro* growth and differentiation studies, and infections of insect vectors, vertebrate hosts, and macrophages. ALD1 ablation lead to diminishment of parasite loads. In host environments, only minor differences were observed in *L*. *mexicana*’s ability to progress normally through its life cycle. Thus, the simplest explanation for the reduced parasitaemia in sand flies and mice infected with ALD1 KO is attenuated fitness (slower growth) that was observed even in axenic culture promastigotes. Considering the strong reduced growth rate phenotype of ALD1 KO, it is plausible that this protein is deeply wired into the metabolic networks of *Leishmania*. This view is corroborated by its ancient acquisition by a common ancestor of Leishmaniinae [18], presumably in the late Cretaceous period [15]. Since this ancestor was a monoxenous trypanosomatid [16] the primordial role of the ancestral *LmxM*.*30*.*2090* homologue may be a broad one relevant for survival in insects, and ALD1 may have retained a similar role. It is important to note that orthologs of *LmxM*.*30*.*2090* are restricted to Leishmaniinae (monoxenous *Crithidia*, *Leptomonas*, *Lotmaria*, *Novymonas*, *Zelonia*, united with dixenous *Leishmania*) and were not found in other trypanosomatids outside of this clade. This implies that ALD1 may have provided evolutionary advantages to the ancestor of Leishmaniinae and allowed it to colonize a wider range of insect hosts by outcompeting slower dividing ALD1-negative kins.

At present it would be premature to discuss the mechanisms governing ALD1’s role in parasite growth and development in hosts. However, since ALD1 shows a global importance for *L*. *mexicana*, identifying the cellular pathway(s) to which it contributes could reveal proteins that are even more vital for basic *L*. *mexicana* function. To that end, biochemical assaying of recombinant ALD1 nucleotide binding and potentially ATP/GTPase activity as well are warranted in the future. Likewise, tagged ALD1 protein pull-down studies to identify binding and interacting proteins would be useful, as some of these may contain domains and motifs that may give a clearer idea of the cellular pathways to which ALD1 contributes. It is possible that future studies will also reveal an explanation for *LmxM*.*30*.*2090* upregulation in amastigote-stage cells [18] that is not evident from this initial characterization.

## Supporting information

S1 Fig Identified regions of interest in ALD1. 382 aa protein encoded by LmxM.30.2090 is depicted with the following regions shown to scale: Walker motif, grey coloration with sub-motifs “A” and “B” designated; H(p), region with strong prediction of helical structure; dotted coloration, region that models to helical domains of various solved structures.
(PPTX)

S2 Fig PCR confirmation of t *LmxM*.*30*.*2090*^-/-^. A, PCR confirmation of correct integration. 1 –Hyg integration into the genomic DNA of KO line is confirmed by PCR with primers SBp_Hyg_f and SBp_Hyg_r (expected size KO 0.3 kb); 2 –Hyg integration confirmed with primers Hyg190_f and Hyg_3’r (expected size KO 0.8 kb); 3 –Hyg replacement of one allele of *LmxM*.*30*.*2090*, primers A and SBp_Hyg_r (expected size KO 1.7 kb); 4 –Sat integration confirmed with primers SBp_SAT_f and SBp_SAT_r (expected size KO 0.3 kb); 5 –*LmxM*.*30*.*2090* gene complete ablation confirmed with primers A and SBp_*LmxM*.*30*.*2090*_r (expected size WT 2 kb); 6 –*LmxM*.*30*.*2090* gene complete ablation confirmed with primers *LmxM*.*30*.*2090*_f and *LmxM*.*30*.*2090*_r (expected size WT 0.25 kb). B, Southern blot analysis of the *Nco* I digested *L*. *mexicana* genomic DNA of the WT, +/- and -/- ALD1 strains with Sat (expected size 4.0 kb), Hyg (expected size 3.4 kb), 5' UTR (expected sizes 4.6, 4.0, and 3.4 kb, respectively), 3' UTR (expected sizes 4.6, 4.0, and 1.2 kb, respectively), and *LmxM*.*30*.*2090* ORF (expected size 4.6 kb) probes. C, Quantitative PCR analysis of the *LmxM*.*30*.*2090* locus in the wild type and ALD1 KO strains. 18S rRNA locus was used for normalization. D, RT-qPCR analysis of the *LmxM*.*30*.*2090* expression. 18S rRNA locus was used for normalization. Data in B and C are from 3 independent biological replicates. See S1 Table for primer sequences.
(PPTX)

S3 Fig Infection in BALB/c mice. Quantitative PCR analysis of the *L*. *mexicana* load in the mouse lesions 13–15 weeks p.i. Boxplots are from two independent biological replicates (4 mice per each group) and show 1st quartile, median, 3rd quartile, and 1.5 x interquartile range values.
(PPTX)

S1 Table List of primers used in this study for amplification of the *LmxM*.*30*.*2090* gene specific targeting sequences, selectable markers, final fusion PCR products used for *L*. *mexicana* transfection, Southern blot probes, and PCR analysis of correct integration. (XLSX)
