# Supplementary figures and images for: A putative ATP/GTP binding protein affects Leishmania mexicana growth in insect vectors and vertebrate hosts
Source: PLoS Negl Trop Dis. 2017 Jul 24;11(7):e0005782. doi: 10.1371/journal.pntd.0005782 (PMC5542692; doi:10.1371/journal.pntd.0005782)

## Slide 1
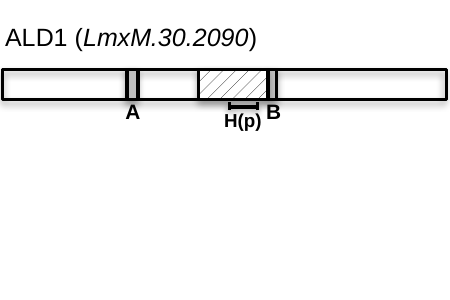

ALD1 (LmxM.30.2090)
A
B
H(p)

Supplement: S1 Fig — 382 aa protein encoded by LmxM.30.2090 is depicted with the following regions shown to scale: Walker motif, grey coloration with sub-motifs “A” and “B” designated; H(p), region with strong prediction of helical structure; dotted coloration, region that models to helical domains of various solved structures. (PPTX) [file pntd.0005782.s001.pptx]

## Slide 1
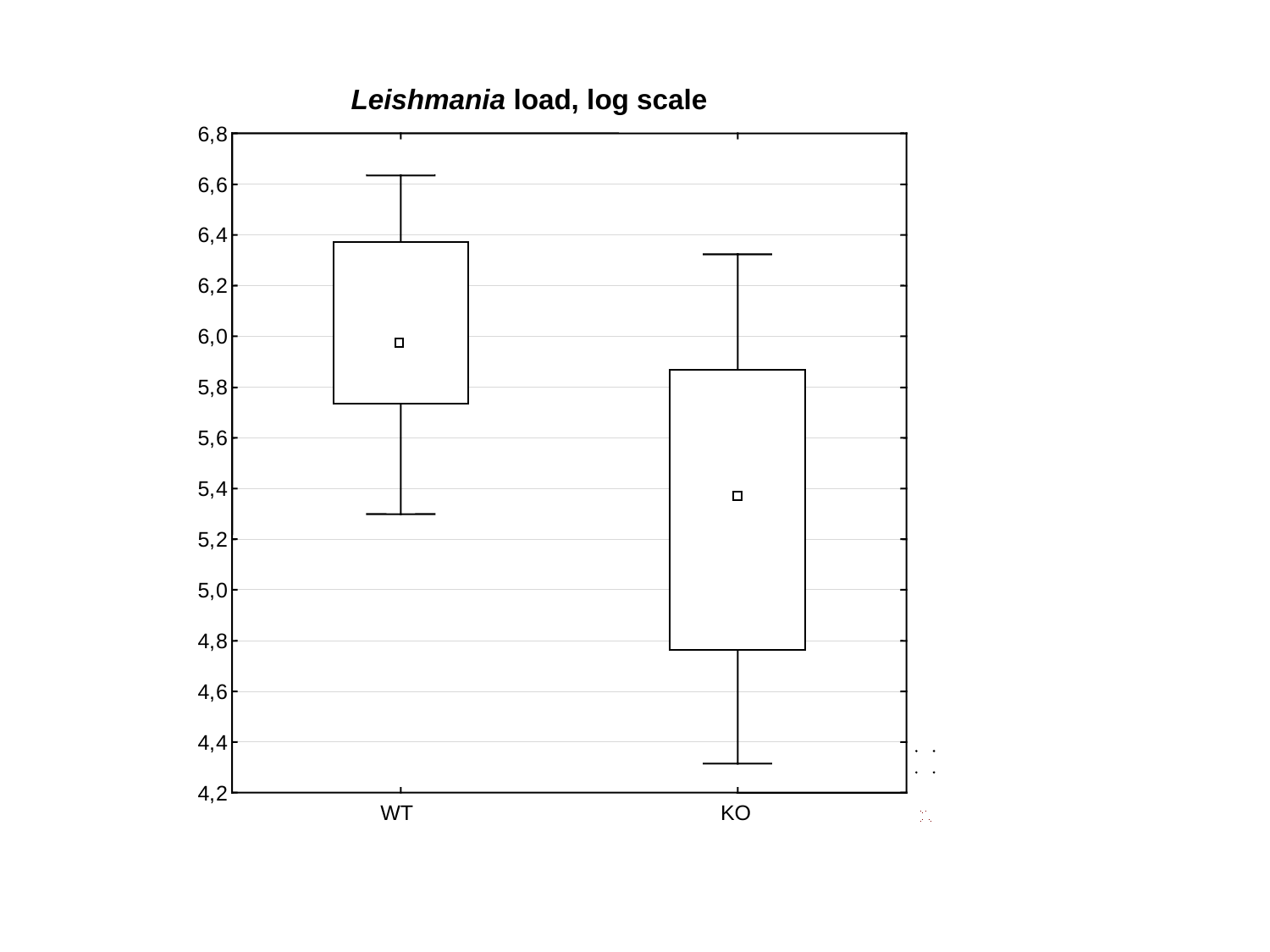

Leishmania load, log scale
6
,
8
6
,
6
6
,
4
6
,
2
6
,
0
5
,
8
5
,
6
5
,
4
5
,
2
5
,
0
4
,
8
4
,
6
4
,
4
4
,
2
WT
KO

Supplement: S3 Fig — Quantitative PCR analysis of the L. mexicana load in the mouse lesions 13–15 weeks p.i. Boxplots are from two independent biological replicates (4 mice per each group) and show 1st quartile, median, 3rd quartile, and 1.5 x interquartile range values. (PPTX) [file pntd.0005782.s003.pptx]
