# Supplementary material for: A putative ATP/GTP binding protein affects Leishmania mexicana growth in insect vectors and vertebrate hosts
Source: PLoS Negl Trop Dis. 2017 Jul 24;11(7):e0005782. doi: 10.1371/journal.pntd.0005782 (PMC5542692; doi:10.1371/journal.pntd.0005782)
Supplement: S2 Fig — A, PCR confirmation of correct integration. 1 –Hyg integration into the genomic DNA of KO line is confirmed by PCR with primers SBp_Hyg_f and SBp_Hyg_r (expected size KO 0.3 kb); 2 –Hyg integration confirmed with primers Hyg190_f and Hyg_3’r (expected size KO 0.8 kb); 3 –Hyg replacement of one allele of LmxM.30.2090, primers A and SBp_Hyg_r (expected size KO 1.7 kb); 4 –Sat integration confirmed with primers SBp_SAT_f and SBp_SAT_r (expected size KO 0.3 kb); 5 –LmxM.30.2090 gene complete ablation confirmed with primers A and SBp_LmxM.30.2090_r (expected size WT 2 kb); 6 –LmxM.30.2090 gene complete ablation confirmed with primers LmxM.30.2090_f and LmxM.30.2090_r (expected size WT 0.25 kb). B, Southern blot analysis of the Nco I digested L. mexicana genomic DNA of the WT, +/- and -/- ALD1 strains with Sat (expected size 4.0 kb), Hyg (expected size 3.4 kb), 5' UTR (expected sizes 4.6, 4.0, and 3.4 kb, respectively), 3' UTR (expected sizes 4.6, 4.0, and 1.2 kb, respectively), and LmxM.30.2090 ORF (expected size 4.6 kb) probes. C, Quantitative PCR analysis of the LmxM.30.2090 locus in the wild type and ALD1 KO strains. 18S rRNA locus was used for normalization. D, RT-qPCR analysis of the LmxM.30.2090 expression. 18S rRNA locus was used for normalization. Data in B and C are from 3 independent biological replicates. See S1 Table for primer sequences. (PPTX) [file pntd.0005782.s002.pptx]

## Slide 1
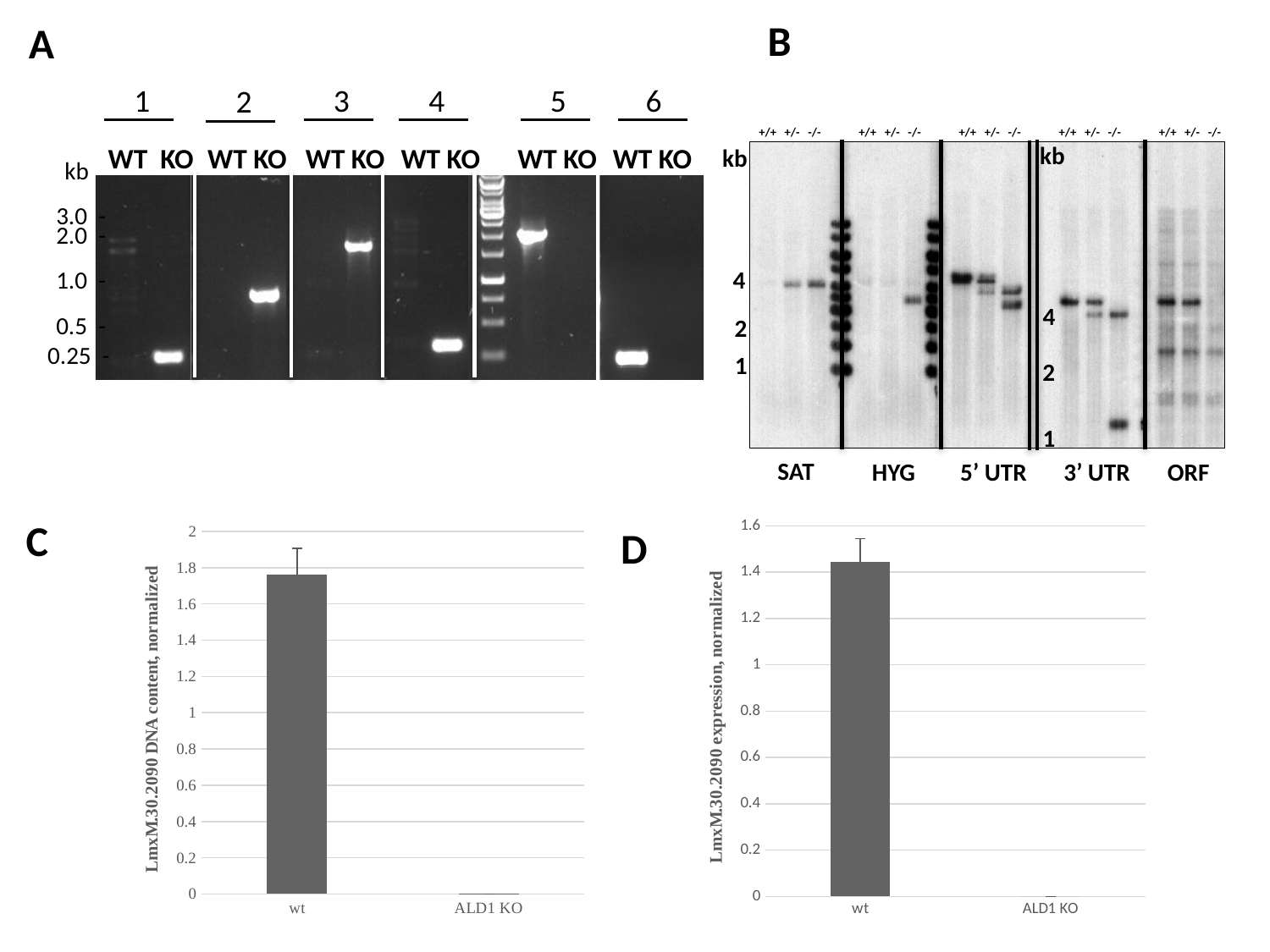

B
A
1
3
4
5
6
2
WT KO
WT KO
WT KO
WT KO
WT KO
WT KO
kb
3.0 -
2.0 -
1.0 -
0.5 -
0.25 -
+/+ +/- -/-
+/+ +/- -/-
+/+ +/- -/-
+/+ +/- -/-
+/+ +/- -/-
kb
kb
4
4
2
1
2
1
SAT
3’ UTR
ORF
HYG
5’ UTR
C
### Chart
| Category | |
|---|---|
| wt | 1.4431118773897529 |
| ALD1 KO | 0.0 |
### Chart
| Category | |
|---|---|
| wt | 1.7626956800457358 |
| ALD1 KO | 1.1231327605806513e-07 |D
